# Secondary Colonic Adenocarcinoma After Continent Cutaneous Urinary Diversion a.m. Lundiana—An Aggressive Disease Entity With High Risk of Locally Advanced Disease and Metastases

**DOI:** 10.1002/iju5.70186

**Published:** 2026-04-28

**Authors:** Fredrik Liedberg, Mats Bläckberg, Johannes Bobjer

**Affiliations:** ^1^ Department of Urology Skåne University Hospital Malmö Sweden; ^2^ Institution of Translational Medicine Lund University Malmö Sweden; ^3^ Department of Urology Helsingborg County Hospital Helsingborg Sweden

**Keywords:** adenocarcinoma, continent cutaneous pouch, Lundiana, urinary diversion

## Abstract

**Introduction:**

We herein report two patients developing secondary adenocarcinomas arising in colonic segments used for urinary diversion a.m. Lundiana in a population‐based series 16 and 27 years after cystectomy, respectively.

**Case Presentation:**

Following initial partial resection of the pouch, both patients experienced local recurrence; one subsequently developed distant metastases and died from secondary adenocarcinoma, whereas the other patient underwent extirpation of the pouch and re‐diversion with an ileal conduit.

**Conclusions:**

Based on these patients, upfront radical surgery would have been a more effective treatment. In this patient population, clinical awareness but not screening for secondary adenocarcinomas seems to be the most appropriate strategy.

## Keynote Message

Secondary adenocarcinomas after continent cutaneous diversion using a colonic segment are rare and occur after a very long time after primary surgery.

## 1 Introduction

Tumor formation in intestinal segments that are transposed to the urinary tract is a long‐term concern for both urologists and patients [1, 2], in addition to more common complications such as metabolic acidosis. The metabolic consequences of exposing intestinal mucosa to urine were recognized nearly a century ago [3]. The highest incidence of secondary adenocarcinomas, with up to one in ten individuals at long‐term follow‐up, has been reported in patients with uro‐colonic anastomoses undergoing ureterosigmoidostomy where urine is mixed with the fecal stream [2]. After bladder augmentation, extrapolation from case series suggests a cumulative long‐term incidence of secondary malignancies (including adenocarcinomas) of up to 5.5% [4]. Secondary colonic adenocarcinomas arising in continent cutaneous pouches constructed from right colonic segments have been reported in case reports and in one systematic review, comprising a total of 19 patients [1]. Herein, we present two additional patients developing adenocarcinomas in continent cutaneous pouches.

## 2 Case Presentation

Two patients operated with cystectomy and urinary diversion with a continent cutaneous pouch a.m. Lundiana constructed from a right colonic and 10‐cm distal ileal segment [5] in a tertial cystectomy unit developed secondary pouch adenocarcinomas at long‐term. Data was ascertained by retrospective chart‐review.

**FIGURE 1:**
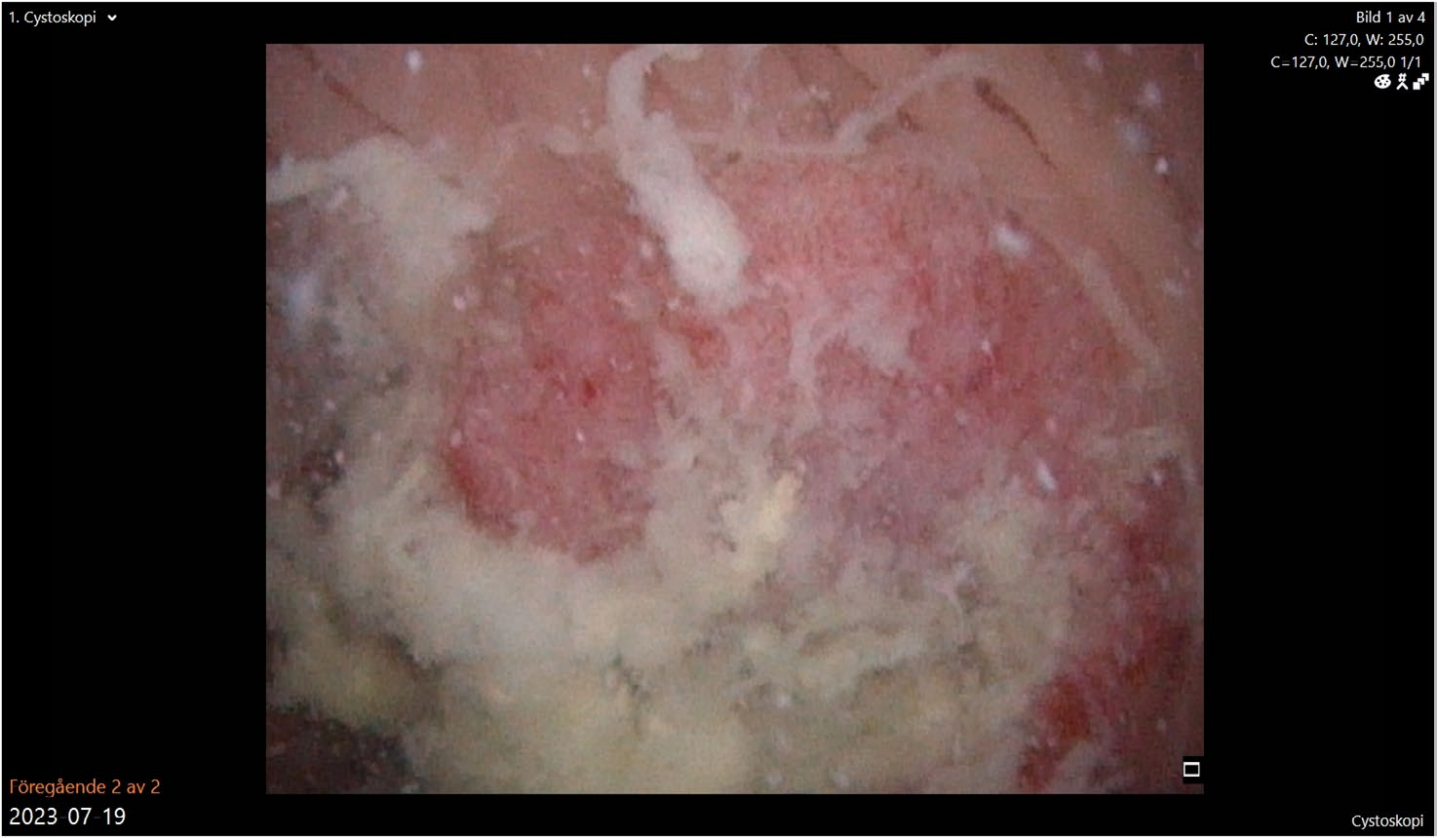
A 3‐cm exophytic tumor as observed at flexible endoscopy of the pouch.

Case 1. A 53‐year old female subjected to radical cystectomy for bladder cancer opted for urinary diversion with a continent cutaneous pouch a.m. Lundiana. Apart from recurrent febrile urinary tract infections that sometimes were associated with right‐sided flank pain, the function of the pouch was good and catheterizations uncomplicated. Twenty‐seven years postoperatively, the patient had macroscopic hematuria, and CT‐urography revealed a 3 cm tumor in the pouch, which was confirmed by endoscopy (Figure 1). The endoscopic biopsy showed dysplastic colonic adenoma. CT thorax was without metastases, and open local excision of the pouch wall including the tumor was performed with macroscopically free margins. The pathological report showed invasive adenocarcinoma extending into pericolic adipose tissue and lymphovascular invasion, however with uncertain microscopic surgical margins. Given the lack of evidence‐based adjuvant treatment options, no such therapy was administered. The patient developed distant (lung metastases) and locoregional tumor recurrence after 6 months. Despite systemic palliative treatment with bevacizumab and fluoropyrimidine/gimeracil/ateracil, radiologic progression occurred. A second systemic palliative line was initiated, yet rapid progression followed and the patient died from disseminated disease shortly thereafter.

**FIGURE 2:**
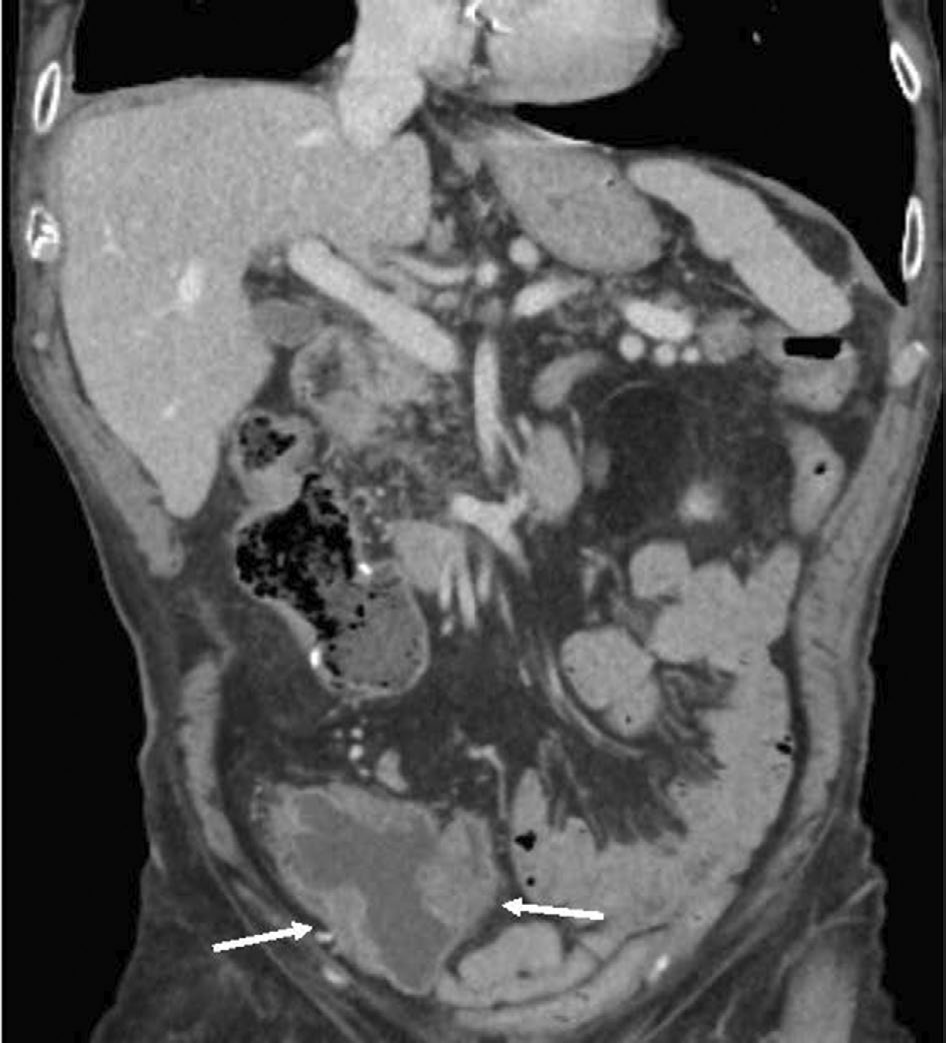
Local recurrences after earlier partial resection (white arrows).

**FIGURE 3:**
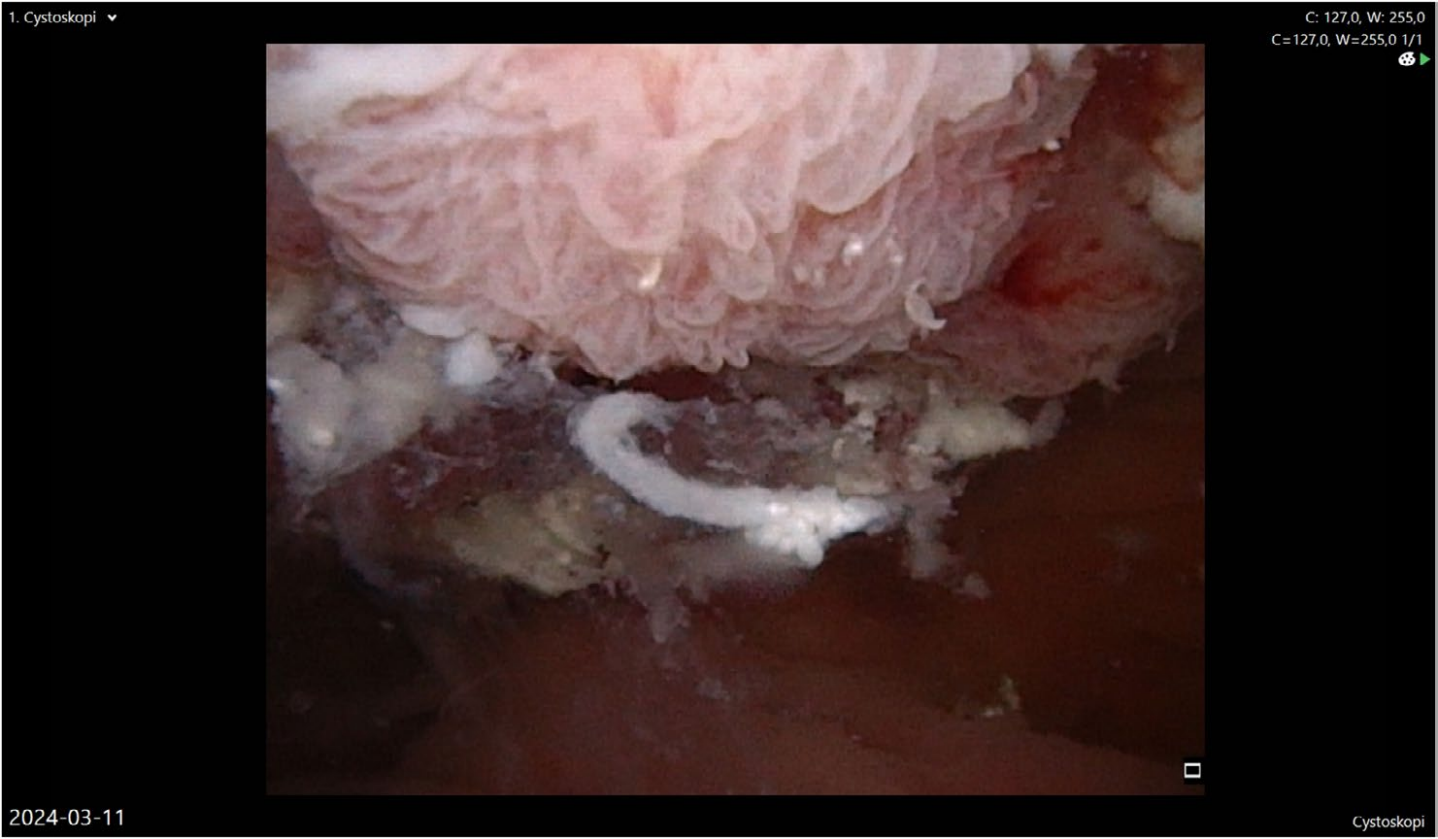
Recurrent secondary adenocarcinoma in the pouch after earlier partial resection as observed at flexible endoscopy of the pouch.

Case 2. A second patient, a 67‐year‐old male, was operated on with cystoprostatectomy for muscle‐invasive bladder cancer and underwent urinary tract reconstruction a.m. Lundiana. Fifteen years later he developed lung cancer and was treated with lobectomy and received adjuvant chemotherapy. During radiological follow‐up after lung cancer treatment, an incidental tumor in the Lundiana pouch was diagnosed. Endoscopic biopsy suggested tubular adenoma with high‐grade dysplasia. Given a tumor diameter of less than 3 cm, the patient was recommended an open resection of the tumor‐bearing portion of the pouch. At surgery an additional tumor was detected despite the preoperative endoscopic evaluation. The pathological report revealed adenocarcinoma infiltrating the musculature of the pouch with uncertain microscopic surgical margins. The second tumor resected displayed only high‐grade dysplasia in a tubulovillous adenoma. When the patient developed a local adenocarcinoma recurrence 2 years later (Figure 2), extirpation of the pouch and rediversion with an ileal conduit was performed. The postoperative course was uneventful. The pathology report revealed locally advanced disease with three adenocarcinoma manifestations, of which the largest (Figure 3) showed transmural tumor growth (pT3) and two metastatic mesenteric lymph nodes. In a shared decision‐making process balancing patient age (85 years) and lack of scientific support for adjuvant treatment, the patient refrained from systemic adjuvant treatment. At last follow‐up 18 months after surgery the patient has no signs of disease recurrence.

## 3 Discussion

**FIGURE 4:**
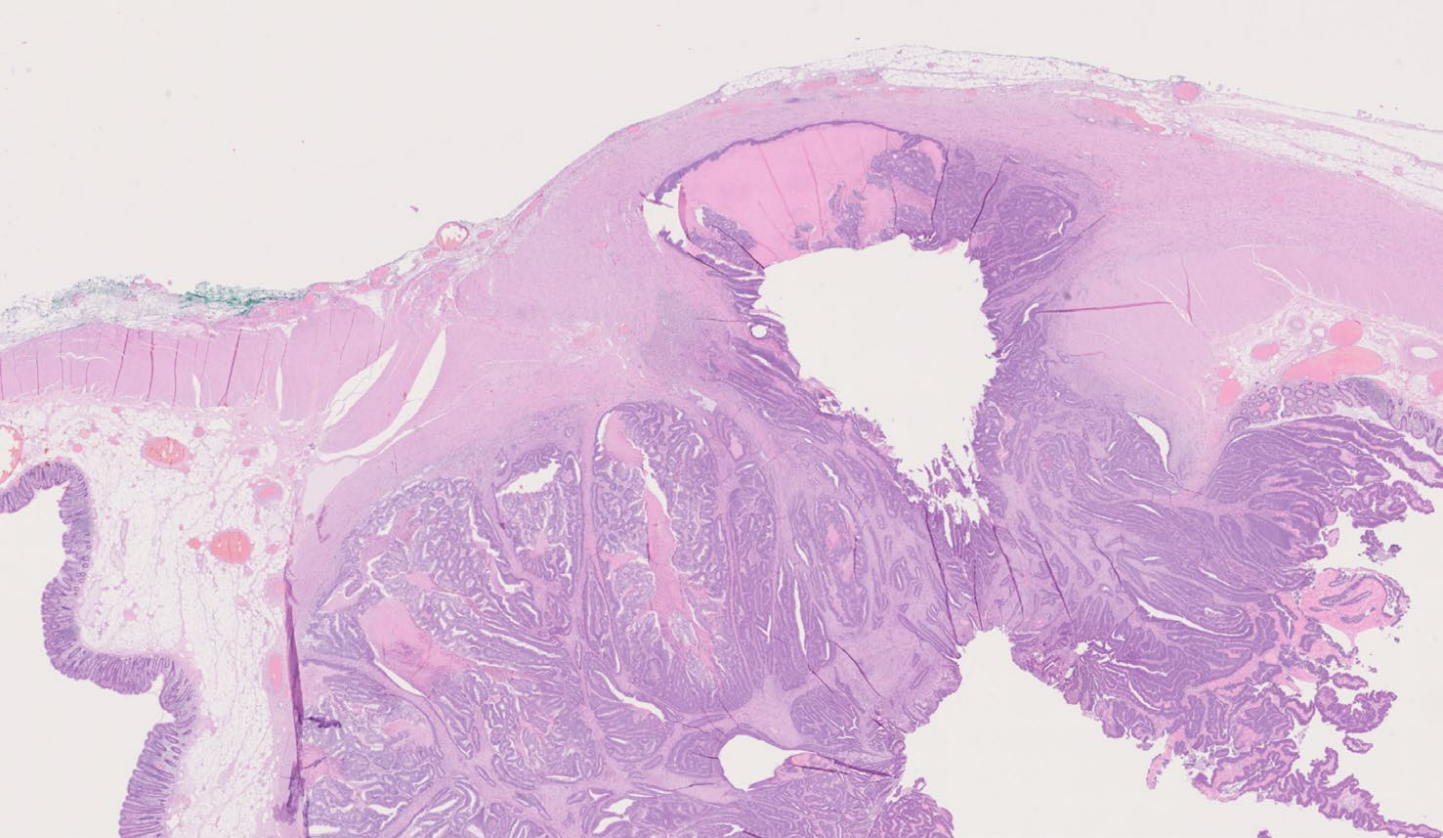
H&E‐staining in Case 1 showing invasion into colonic segment.

This case‐report describes a rare entity of secondary adenocarcinomas in a Lundiana pouch detected 27 and 16 years after radical cystectomy, respectively. Both patients had been included in a population‐based third‐party assessment of long‐term complications among 193 patients who underwent Lundiana pouch reconstruction [5]. However, these recurrences were not captured in that study as they occurred at a later timepoint. Both patients had pure urothelial carcinomas in the cystectomy specimens without adenocarcinomas or glandular differentiation. Thus, the pouch‐adenocarcinomas originated from the colonic segment (Figure 4). The fact that both patients underwent one unsuccessful attempt at diversion‐preserving surgery and subsequently suffered from local recurrence suggests that such an approach should be considered with caution. Although endoscopic resection has been proposed for selected patients in the literature, these recommendations are based on case reports only [6]. Endoscopic biopsies in both patients showed only high‐grade dysplasia, despite that advanced disease stage was observed in the resected specimens. This underscores the risk of underestimating disease extent and supports the notion that radical surgery may be a safer option compared to endoscopic or partial resection of a continent cutaneous pouch with secondary adenocarcinoma. This aligns with the findings from a systematic review, in which half of the patients were diagnosed at an advanced stage [1]. Nonetheless, when considering re‐diversions after urinary diversion, the substantial risk of further re‐operations, reported in more than every second patient related to malfunction of the re‐reconstructed urinary tract, must be weighed against the oncological need for radical excision of secondary adenocarcinomas [7].

The rarity of secondary adenocarcinomas after continent cutaneous diversion using colonic segments makes any recommendations for surveillance endoscopies difficult to motivate. Attempts to explore the use of serum carcinoembryonic antigen (CEA) as a tumor marker in this setting have shown that elevated CEA is a common finding in patients with urinary diversions, and consequently not useful as a surveillance method for secondary adenocarcinomas [8]. Still, in particular settings such as individuals with Lynch Syndrome and urinary diversion, formal endoscopic surveillance of a continent cutaneous diversion might be adequate (9).

The current case report and existing literature suggest that local symptoms such as macroscopic hematuria, also at long‐term, should not be neglected in patients with continent cutaneous pouches constructed from colonic segments. It seems reasonable to perform flexible endoscopy of the pouch in addition to CT‐urography at such occasions, also for the possibility to assess possible benign differential diagnoses such as upper tract stones and pouch stones. Thus, information about the possibility of secondary adenocarcinomas to patients and caregivers is essential to convey the consequences of local symptoms from continent pouches also at very long‐term.

Our experience points towards recommending primary extirpation of the pouch and re‐diversion as an oncologically safer approach, rather than considering local resection in patients with secondary adenocarcinomas in continent cutaneous pouches constructed from colonic segments. When considering local resection, it appears essential to ensure a radical resection with clear microscopic margins as in Case 1, and confirm the absence of multifocal tumor manifestations suggestive of a field disease. In the multidisciplinary decision‐making process choosing between local resection or complete extirpation of the pouch, additional considerations include involving a colorectal cancer specialist and allowing for maintaining a sufficient residual pouch volume after a local resection. Awareness of the disease entity and the possibility of disease development occurring a very long time after primary surgery needs to be conveyed to patients and caregivers.

## Funding

This work was supported by the Swedish Cancer Society (CAN 2023/2807), Swedish Research Council (2021–00859), Lund Medical Faculty (ALF), Skåne University Hospital Research Funds, Maud and Birger Gustavsson Foundation, Hjelm Foundation, The Cancer Research Fund at Malmö General Hospital, Skåne County Council's Research and Development Foundation (REGSKANE‐622351), Gösta Jönsson Research Foundation, the Foundation of Urological Research (Ove and Carin Carlsson bladder cancer donation and Astrid and Roland Bengtsson upper tract urothelial carcinoma donation), Sjöberg Foundation, Hillevi Fries Research Foundation. The funding sources had no role in the study design, data analyses, interpretation of the results, or writing of the manuscript.

## Ethics Statement

Approval by Research Ethics Board of Lund University, Sweden (Dnr 2023–04696‐02 and Ö 49–2023/3.1).

## Consent

The authors have nothing to report.

## Conflicts of Interest

The authors declare no conflicts of interest.

## Data Availability

The data that support the findings of this study are available on request from the corresponding author. The data are not publicly available due to privacy or ethical restrictions.
